# Betulinic acid inhibits colon cancer cell and tumor growth and induces proteasome-dependent and -independent downregulation of specificity proteins (Sp) transcription factors

**DOI:** 10.1186/1471-2407-11-371

**Published:** 2011-08-24

**Authors:** Sudhakar Chintharlapalli, Sabitha Papineni, Ping Lei, Satya Pathi, Stephen Safe

**Affiliations:** 1 Institute of Biosciences and Technology, Texas A&M Health Science Center, Houston, TX 77030 USA; 2 Department of Veterinary Physiology & Pharmacology, Texas A&M University, College Station, TX 77843 USA; 3 Eli Lilly Co., Oncology Division, Indianapolis, IN, USA; 4 Dow Agrosciences, Indianapolis, IN, USA

## Abstract

**Background:**

Betulinic acid (BA) inhibits growth of several cancer cell lines and tumors and the effects of BA have been attributed to its mitochondriotoxicity and inhibition of multiple pro-oncogenic factors. Previous studies show that BA induces proteasome-dependent degradation of specificity protein (Sp) transcription factors Sp1, Sp3 and Sp4 in prostate cancer cells and this study focused on the mechanism of action of BA in colon cancer cells.

**Methods:**

The effects of BA on colon cancer cell proliferation and apoptosis and tumor growth *in vivo *were determined using standardized assays. The effects of BA on Sp proteins and Sp-regulated gene products were analyzed by western blots, and real time PCR was used to determine microRNA-27a (miR-27a) and ZBTB10 mRNA expression.

**Results:**

BA inhibited growth and induced apoptosis in RKO and SW480 colon cancer cells and inhibited tumor growth in athymic nude mice bearing RKO cells as xenograft. BA also decreased expression of Sp1, Sp3 and Sp4 transcription factors which are overexpressed in colon cancer cells and decreased levels of several Sp-regulated genes including survivin, vascular endothelial growth factor, p65 sub-unit of NFκB, epidermal growth factor receptor, cyclin D1, and pituitary tumor transforming gene-1. The mechanism of action of BA was dependent on cell context, since BA induced proteasome-dependent and proteasome-independent downregulation of Sp1, Sp3 and Sp4 in SW480 and RKO cells, respectively. In RKO cells, the mechanism of BA-induced repression of Sp1, Sp3 and Sp4 was due to induction of reactive oxygen species (ROS), ROS-mediated repression of microRNA-27a, and induction of the Sp repressor gene ZBTB10.

**Conclusions:**

These results suggest that the anticancer activity of BA in colon cancer cells is due, in part, to downregulation of Sp1, Sp3 and Sp4 transcription factors; however, the mechanism of this response is cell context-dependent.

## Background

Colorectal cancer is a leading cause of death in most developed countries including the United States, and in 2010 it is estimated that over 102,700 new cases will be diagnosed and 51,370 deaths will occur in the United States [1]. Genetic susceptibility accounts for 15 - 25% of colon cancer cases, and genetic markers provide important insights on factors important for the molecular and genetic changes that result in development of this disease [2]. Familial adenomatous polyposis syndromes [3,4], hereditary non-polyposis colorectal cancer [5-8], and other polyposis syndromes which increase the incidence of colorectal cancer including Peutz Jegher's syndrome, familial juvenile polyposis, and hereditary mixed polyposis syndrome, are linked to mutations in LKB1, STK11, SMAD4, PTEN, E-cadherin, cyclin D1, and transforming growth factor β receptors [2].

The incidence rates of sporadic colon cancer are highly variable among different regions of the world and the changes in incidence of this disease in migrants suggests that environmental factors related to diet contribute to development of colon cancer [9,10]. Fruits, nuts and vegetables contain diverse anticarcinogenic phytochemicals; however, epidemiological studies give variable results with respect to their chemopreventive effects and similar variability among studies has been reported for the protective effects of dietary folate [11-14]. Most colon cancer patients present with localized disease which is treated with curative surgery; however, disease relapse is experienced by up to 40% of patients [15-17]. Cytotoxic drugs are primarily used for colon cancer chemotherapy and there is a increasing need to develop mechanism-based drugs for treating this disease.

Specificity protein (Sp) transcription factors Sp1, Sp3 and Sp4 are overexpressed in colon and other cancer cell lines [18-23], and Sp1 is a negative prognostic factor for survival of pancreatic and gastric cancer patients [24,25]. The potential importance of Sp transcription factors as drug targets is due not only to their overexpression in multiple tumor types but also to their relatively low expression in non-tumor rodent and human tissues, and this is consistent with the reported decrease of Sp1 expression with increasing age [26-28]. RNA interference studies which knockdown Sp1, Sp3 and Sp4 (individually or combined) have identified several Sp-regulated gene-products that are themselves individual targets for new mechanism-based drugs. Sp-regulated genes include several that are important for cancer cell proliferation [cyclin D1, epidermal growth factor receptor (EGFR), hepatocyte growth factor receptor (c-MET)], survival (bcl-2 and survivin), angiogenesis [vascular endothelial growth factor (VEGF) and its receptors (VEGFR1/R2) and pituitary tumor-transforming gene 1 (PTTG-1)], and inflammation (p65 subunit of NFκB) [23,29-38].

Betulinic acid (BA) is a naturally occurring triterpenoid which inhibits growth of multiple tumors [39,40]. Studies in this laboratory show that BA inhibits prostate cancer cell and tumor (xenograft) growth and this is due, in part, to proteasome-dependent downregulation of Sp1, Sp3, Sp4 and several Sp-regulated genes [20]. In this study, we show that BA inhibits growth of colon cancer cells and tumors and downregulates Sp transcription factors through activation of proteasome-dependent (SW480 cells) and proteasome-independent (RKO cells) pathways.

## Methods

### Cell proliferation and cell cycle progression assays

The RKO and SW480 colon cancer cell lines were previously characterized at the M.D. Anderson Cancer Center (Houston, TX) and kindly provided by Dr. Stanley Hamilton. RKO and SW480 colon cancer cells (2 × 10^4 ^per well) were plated in 12-well plates and allowed to attach for 24 h. The medium was then changed to DMEM/Ham's F-12 medium containing 2.5% charcoal-stripped FBS, and either vehicle [dimethyl sulfoxide (DMSO)] or different concentrations of the compound were added. Fresh medium and compounds were added every 48 h, and cells were then trypsinized and counted after 48 and 96 h using a Coulter Z1 cell counter. Results are expressed as means ± SE for at least 3 replicate determinations for each treatment group. RKO and SW480 cells were treated with either the vehicle (DMSO) or BA for 24 h. Cells were trypsinized, centrifuged and resuspended in staining solution containing 50 μg/ml propidium iodide, 4 mmol/L sodium citrate, and 30 units/ml RNase. After incubation at room temperature for 1 h, cells were analyzed on a FACS Vantage SE DiVa made by Becton Dickinson, using FACSDiva Software V4.1.1. Propidium iodide (PI) fluorescence was collected through a 610SP bandpass filter, and list mode data were acquired on a minimum of 50,000 single cells defined by a dot plot of PI width *vs*. PI area. Data analysis was performed in BD FACSDiva Software V4.1.1 using PI width *vs*. PI area to exclude cell aggregates.

### Plasmids, transfection assay and antibodies

Sp1 and Sp3 promoter constructs were kindly provided by Drs. Carlos Cuidad and Veronique Noe (University of Barcelona, Barcelona, Spain). The pVEGF-2068 construct contains a VEGF promoter insert (positions -2068 to +54) linked to luciferase reporter gene. The pSurvivin-269 was kindly provided by Dr. M. Zhou (Emory University, Atlanta, GA). The PTTG-1-luc construct containing the -1373 to +3 region of the PTTG-1 promoter was provided by Dr. Kakar (University of Louisville, Louisville, KY). Colon cancer cells (1.5 × 10^5^) were seeded in 12-well plates using DMEM:Ham's F-12 media containing 2.5% charcoal stripped serum. After 24 h, cells were transfected with 0.4 μg of reporter gene constructs and 0.04 μg of β-Gal using Lipofectamine 2000 according to manufacturer's protocol. Reporter lysis buffer and luciferase reagent for luciferase studies were supplied by Promega (Madison, WI). Five h after transfection, cells were treated with control or BA for 22-24 h and luciferase activity (normalized to β-galactosidase) was determined using Lumicount luminometer (PerkinElmer Life and Analytical Sciences). For RNA interference assays with iSp, a mixture of oligonucleotides containing siRNAs against Sp1, Sp3 and Sp4 (combined) was used as previously described [20,21,34]. Antibodies for Sp1, Sp3, Sp4, cyclin D1, EGFR, NFκB (p65), VEGF and VEGFR1 were purchased from Santa Cruz Biotechnology (Santa Cruz, CA). c-PARP and survivin antibodies were purchased from Cell Signaling Technology (Danvers, MA). Monoclonal β-actin antibody was purchased from Sigma-Aldrich. Western blots were determined with whole cell lysates essentially as described [20-23].

### Northern blot analysis

For miRNA analysis, 20 μg total RNA per lane was electrophoresed on 15% TBE urea polyacrylaminde gel (Invitrogen), electrophoretically transferred in 0.5 × TBE at 300 mÅ for 45 min to GeneScreen Plus membrane (PerkinElmer, Boston, MA), UV cross-linked and hybridized in ULTRAhyb-Oligo hybridization buffer (Ambion, Austin, TX) at 42°C with ^32^P end-labeled DNA oligonucleotides complementary to miR-27a. Blots were washed at 42°C in 2X SSC and 0.5% SDS for 30 min with gentle agitation.

### Semiquantitative reverse transcription and real time PCR

RKO and SW480 colon cancer cells were treated with BA at different concentrations for 24 h. Total RNA was extracted using RNeasy Mini Kit (Qiagen), and 2 μg of RNA was used to synthesize cDNA using Reverse Transcription System (Promega). Primers were obtained from IDT and used for amplification were as follows: ZBTB10 (sense 5'-GCT GGA TAG TAG TTA TGT TGC-3'; antisense 5'-CTG AGT GGT TTG ATG GAC AGA G-3'). PCR products were electrophoresed on 1% agarose gels containing ethidium bromide and visualized under UV transillumination. Real time PCR for determining miR-27a, ZBTB10 and Myt-1 RNA levels were determined essentially as described [20-23].

### Reactive oxygen species (ROS) and mitochondrial membrane potential assays

Cellular ROS levels were evaluated with the cell permeant probe CM-H_2_DCFDA (5-(and-6)-chloromethyl-2'7'-dichlorodihydrofluorescein diacetate acetyl ester) from Invitrogen. Following 36 h treatment, cells plated on a 6-well cell culture plate were loaded with 10 μM CM-H_2_DCFDA for 1 h, washed once with serum-free medium, and analyzed for ROS levels using Beckman Coulter XL four-color cytometer. Each experiment was done in triplicate and results are expressed as mean ± S.E. for each treatment group. Mitochondrial membrane potential (MMP) was measured with Mitochondrial Membrane Potential Detection Kit (Stratagene) according to the manufacturer's protocol using JC-1 dye, and mitochondrial membrane potential shift was measured using FACS Calibur flow cytometer using CellQuest acquisition software (BD Biosciences). J-aggregates were detected as red fluorescence and J-monomers are detected as green fluorescence.

### Xenograft studies in athymic mice

Female athymic nude mice were purchased from Harlan Laboratories (Indianapolis, IN) and were cared for and used in accordance with institutional guidelines. To produce tumors, RKO cells (5 × 10^6^; ≥ 90% viable) were subcutaneously injected into the flanks of individual mice. Tumors were allowed to grow for 6 days until palpable and mice were then randomized into two groups (6 mice/group) and dosed by oral gavage with corn oil or BA (25 mg/kg/day) every second day for 22 days. The mice were weighed, and tumor size was measured every second day with calipers to permit calculation of tumor volumes: V = LW^2^/2, where L and W were length and width, respectively. After BA treatment, the animals were sacrificed; final body and tumor weights were determined, and major visceral organs were collected and lysates were used for western blot analysis of Sp proteins.

## Results

### 1. BA inhibits colon cancer cell growth and induces apoptosis

**Figure 1:**
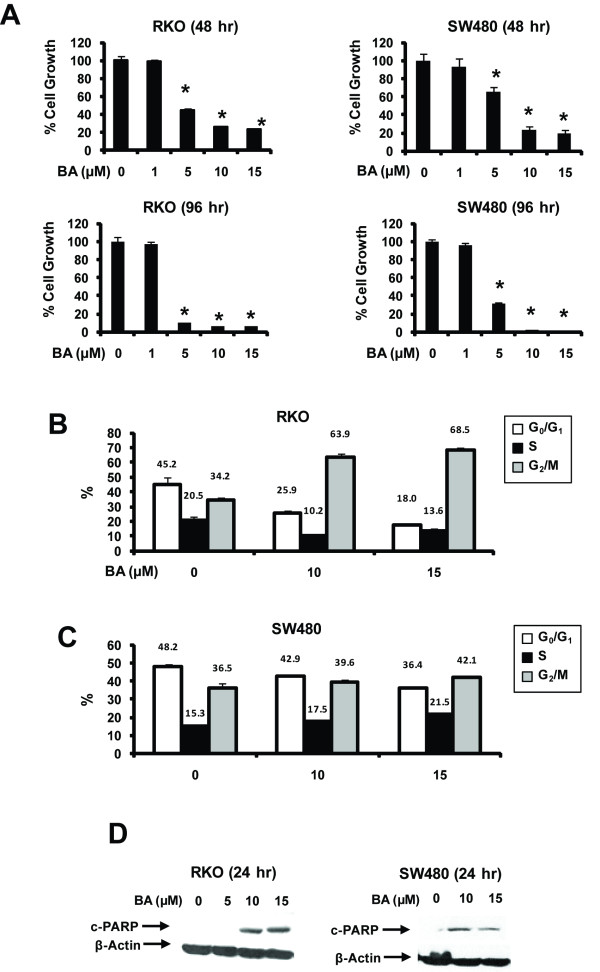
**BA inhibits growth and induces apoptosis in colon cancer cells**. (A) Inhibition of cell proliferation. Cells were treated with BA for 48 or 96 h and then counted as described in the Materials and Methods. Cell cycle progression in RKO (B) and SW480 (C) cells. Cells were treated with DMSO (0), 10 or 15 μM BA for 24 h and analyzed by FACS analysis as described in the Materials and Methods. (D) Induction of PARP cleavage. RKO and SW480 cells were treated with BA for 24 h and whole cell lysates were analyzed for cleaved PARP as described in the Materials and Methods. Results in (A) - (C) are expressed as means ± SE for at least 3 replicate experiments and significant (p < 0.05) differences from controls (0, DMSO) are indicated (*).

BA inhibits growth of multiple cancer cell lines [39,40], and results in Figure 1A demonstrate that BA inhibited RKO and SW480 cell proliferation after treatment for 48 or 96 h. Growth inhibition was observed at concentrations of ≥ 5 μM in both cell lines at the two time points. The effects of BA on distribution of cells in G_0_/G_1_, S and G_2_/M phases of the cell cycle was cell context-dependent (Figures 1B and 1C). In RKO cells, BA dramatically decreased the percentage of cells in G_0_/G_1 _and S phase and increased the percent in G_2_/M, whereas in SW480 cells, there was a slight decrease in G_0_/G_1 _and S phase and a parallel increase in the percentage of cells in G_2_/M. Treatment of RKO and SW480 cells with BA also enhanced PARP cleavage (Figure 1D) which is consistent with induction of apoptosis in these cell lines; however, after treatment of the cells for only 24 hr, < 10% of the cells were sub-G_1 _in FACS analysis.

### 2. BA decreases expression of Sp1, Sp3, Sp4 and Sp-regulated gene products in colon cancer cells

**Figure 2:**
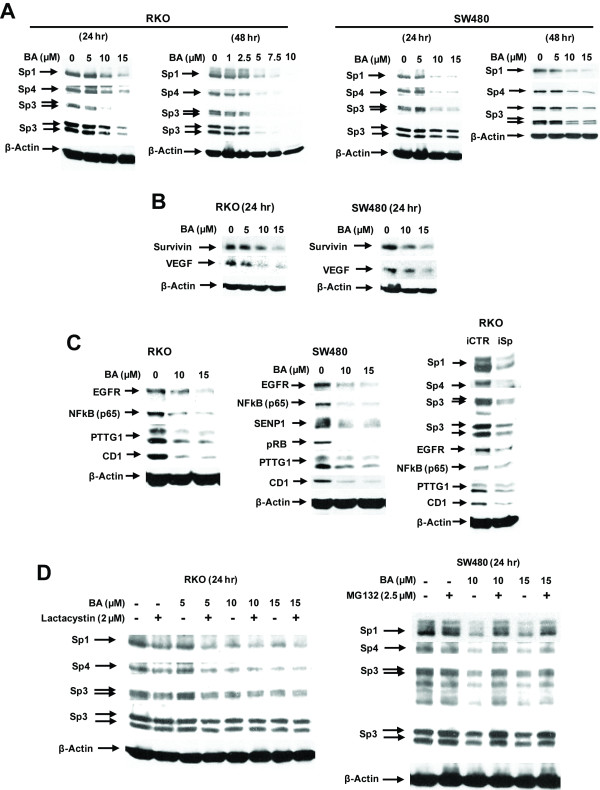
**BA decreases Sp proteins and Sp-regulated genes**. BA decreases Sp proteins in RKO and SW480 cells (A) and Sp-regulated genes (B). Cells were treated with DMSO (0) or BA for the indicated times and whole cell lysates were analyzed by western blots as described in the Materials and Methods. (C) BA and Sp1/Sp3/Sp4 (iSp) knockdown decrease Sp-regulated genes. Cells were either treated with BA or transfected with iSp and whole cell lysates were analyzed by western blots as described in the Materials and Methods. (D) Effects of proteasome inhibitors. Cells were treated with BA ± proteasome inhibitors MG132 or lactacystin for 24 h and whole cell lysates were analyzed by western blots as described in the Materials and Methods.

Previous studies showed that BA decreases Sp1, Sp3 and Sp4 protein expression in prostate and bladder cancer cells [20,32], and results in Figure 2A confirm that similar effects were observed in RKO and SW480 cells. These results were similar to that observed for CDODA-Me and GT-094 in these cell lines [38,40]. Moreover, BA also induced cleaved PARP and decreased expression of survivin, an inhibitor of apoptosis in RKO and SW480 cells, and VEGF (Figure 2B). These results are consistent with previous studies showing that both survivin and VEGF are Sp-regulated genes [20,29]. RNA interference studies which knockdown Sp1, Sp3 and Sp4 (individually or combined) have identified EGFR, p65 subunit of NFκB, PTTG1 and cyclin D1 as Sp-regulated genes [32-35], and results in Figure 2C show that BA decreased expression of their corresponding gene products in RKO and SW480 cells. Moreover combined knockdown of Sp1, Sp3 and Sp4 using an oligonucleotide cocktail (iSp) [20,21,32-34] also decreased expression of p65, PTTG-1, EGFR and cyclin D1 in RKO cells (Figure 2C) confirming their regulation by Sp transcription factors in colon cancer cells.

Treatment of RKO cells with the proteasome inhibitor MG132 alone was cytotoxic; however, in RKO cells treated with BA or the proteasome inhibitor lactacystin alone or in combination, BA-induced downregulation of Sp1, Sp3, and Sp4 was not inhibited indicating that the effects were proteasome-independent (Figure 2D). MG132 was not toxic to SW480 cells and BA-induced downregulation of Sp1, Sp3 and Sp4 was reversed in cells cotreated with BA plus MG132 (and lactacystin; data not shown), demonstrating a proteasome-dependent pathway in this cell line as previously observed in LNCaP cells treated with BA [20].

### 3. BA decreases Sp and Sp regulated gene expression in RKO cells through disruption of miR-27a:ZBTB10

**Figure 3:**
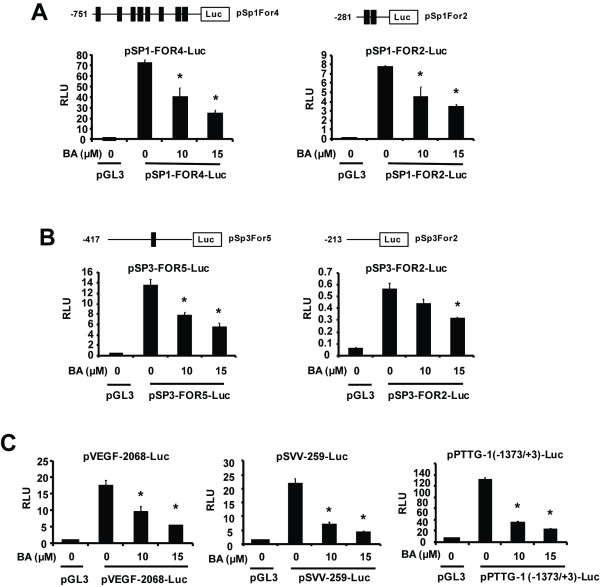
**BA inhibits luciferase activity in RKO cells transfected with GC-rich constructs**. Transfection with constructs containing Sp1 (A), Sp3 (B) and Sp-regulated (C) gene promoter constructs. RKO cells were transfected with the indicated constructs, treated with DMSO or BA, and luciferase activity (normalized to β-galactosidase) was determined as described in the Materials and Methods. Results are expressed as means ± SE for at least 3 replicated determinations as significant (p < 0.05) is indicated (*).

We further investigated BA-mediated repression of Sp and Sp-regulated genes in RKO cells by determining the effects of BA on a series of GC-rich constructs containing promoter inserts from the Sp1, Sp3, VEGF, survivin and PTTG-1 which are downregulated after loss of Sp proteins [18-21,29,30]. Results in Figures 3A and 3B show that BA decreased luciferase activity in RKO cells transfected with pSp1-FOR4-luc, pSp1-FOR2-luc, pSp3-FOR5-luc and pSp3-FOR2-luc constructs which contain the GC-rich -751 to -20 and -281 to -20 region of the Sp1 gene promoter and the GC-rich -417 to -38 and -213 to -38 regions of the Sp3 promoter, respectively [36]. BA also decreased luciferase activity in RKO cells transfected constructs containing VEGF (-2018 to +5), survivin (-259 to +49), and PTTG-1 (-1373 to +3) promoter inserts (Figure 3C), and these results are also consistent with previous studies using agents or RNA interference that downregulate Sp protein expression [19,20,30].

**Figure 4:**
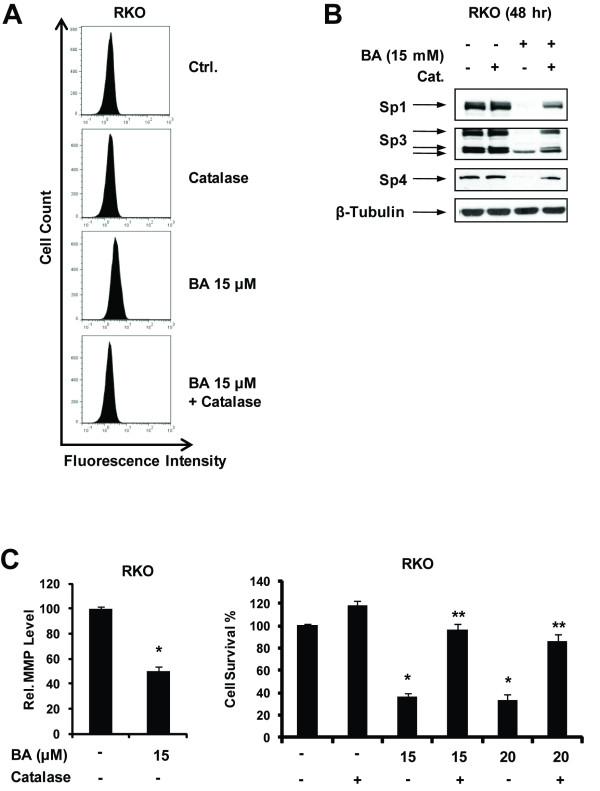
**BA decreases MMP and induces ROS in RKO cells**. (A) Induction of ROS. RKO cells were treated with 15 μM BA, catalase or BA plus catalase for 36 h and ROS production was determined using the fluorescent probe H2DCFDA as described in the Materials and Methods. Role of ROS in BA-induced Sp downregulation (B) and growth inhibition and BA effects on MMP (C). Cells were treated with DMSO (control), BA, catalase or BA plus catalase and Sp proteins (in whole cell lysates), MMP and cell growth were determined as described in the Materials and Methods. Results (C) are expressed as means ± SE for 3 replicate experiments and significant (p < 0.05) growth inhibition by BA (*) and rescue by catalase (**) are indicated. BA-induced inhibition of MMP was not affected by cotreatment with catalase (data not shown).

ROS and hydrogen peroxide (H_2_O_2_) play a role in downregulation of Sp1, Sp3 and Sp4 in pancreatic and bladder cancer cells [34,37] and treatment of RKO cells with BA for 36 h induced ROS as determined by FACS analysis using the fluorescent ROS scavenger H_2_DCFDA (Figure 4A). Moreover, in cells treated with BA plus catalase, there was a decrease in fluorescence indicating that catalase inhibited ROS formation. Treatment of RKO cells with BA decreased expression of Sp1, Sp3 and Sp4 proteins and this effect was partially reversed in RKO cells cotreated with BA plus catalase (Figure 4B). BA-decreased MMP was indicated by increased green/red fluorescence associated with the JC-1 monomer and aggregates, respectively; moreover, BA-induced growth inhibition was also reversed in RKO cells cotreated with BA plus catalase (Figure 4C), thus confirming an important role for ROS (H_2_O_2_) in mediating the growth inhibitory effects of BA.

**Figure 5:**
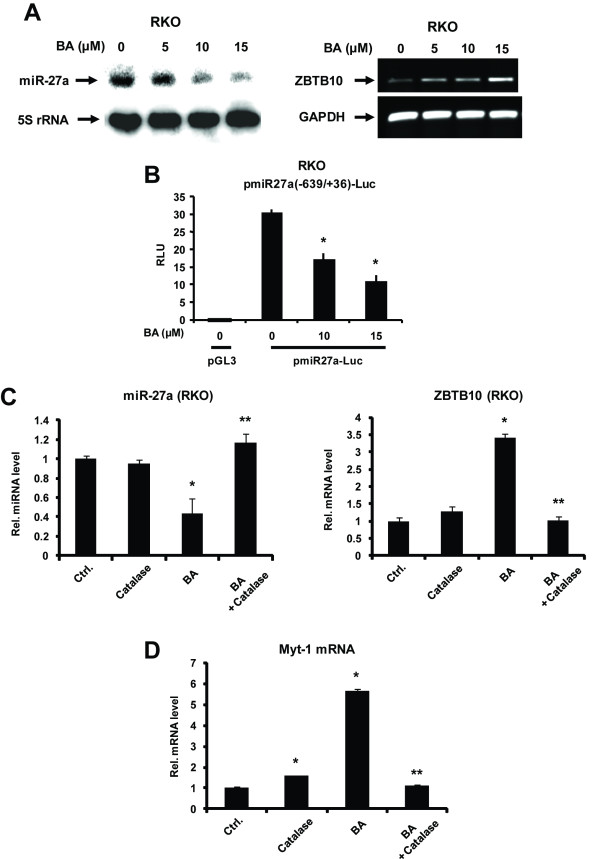
**Role of miR-27a in regulation of BA-mediated responses**. (A) Repression of miR-27a and induction of ZBTB10. RKO cells were treated with DMSO or BA and miR-27a and ZBTB10 expression were determined by Northern blot and semi-quantitative RT-PCR, respectively, as described in the Materials and Methods. (B) BA decreases miR-27a promoter activity. RKO cells were transfected with pMiR-27a(-639/+36)-luc, treated with DMSO or BA and luciferase activity determined as described in the Materials and Methods. Role of BA-induced ROS on expression of miR-27a and ZBTB10 (C) and Myt-1 (D). RKO cells were treated with DMSO, BA, catalase or BA plus catalase for 36 h and miR-27a, ZBTB10 and Myt1 mRNA levels were determined by real time PCR as described in the Materials and Methods. Results in (B) - (D) are expressed as means ± SE for at least 3 replicate determinations and significant (p < 0.05) effects by BA (*) and reversal by catalase (**) are indicated.

Previous studies show that ROS-dependent disruption of miR-27a:ZBTB10 is important for Sp downregulation [33,38] and Figure 5A shows that BA decreased miR-27a, as determined by Northern blot analysis, and semi-quantitative RT-PCR confirmed induction of ZBTB10. Moreover, downregulation of miR-27a was also paralleled by decreased luciferase activity in RKO cells transfected with a construct (pmiR-27a-luc) containing the -639 to +36 region of the promoter for the miR-23a-miR-27a-miR-24-2 [41] cluster (Figure 5B). Using real time PCR, BA significantly decreased miR-27a and induced ZBTB10 (Figure 5C) expression and these responses were all significantly attenuated in RKO cells cotreated with BA plus catalase. These results confirm that BA-induced suppression of Sp1, Sp3 and Sp4 is linked to induction of ROS and ROS-mediated disruption of miR-27a:ZBTB10. The Myt-1 gene is associated with G_2_/M arrest and is repressed by miR-27a in colon and breast cancer cells [22,36]. BA induced Myt-1 mRNA in RKO cells; this response was also attenuated in cells cotreated with BA plus catalase (Figure 5D) and this was consistent with ROS-mediated regulation of miR-27a and ZBTB10 (Figure 5C).

### 4. BA inhibits colon tumor growth

**Figure 6:**
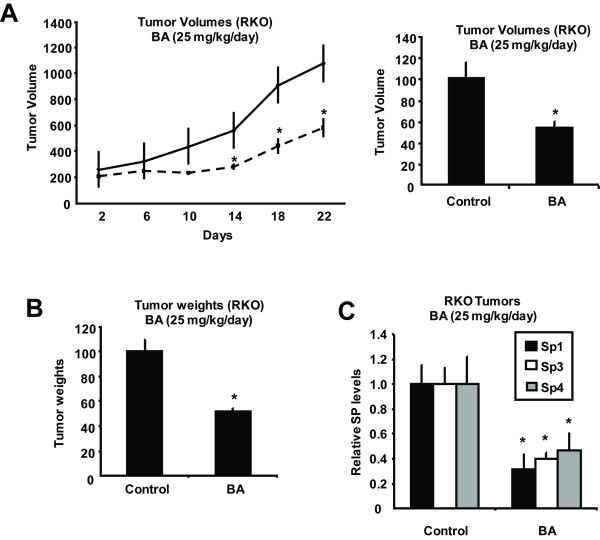
**BA inhibits colon tumor growth *in vivo***. Inhibition of tumor growth (A) and weights (B). BA (25 mg/kg/d) was administered (orally) to athymic nude mice every second day and tumor volumes and weights were determined as described in the Materials and Methods. (C) Downregulation of Sp1, Sp3 and Sp4. Tumor lysates from individual mice were analyzed by western blots as described in the Materials and Methods and Sp1, Sp3 and Sp4 protein levels were normalized to β-actin. Results in (A) - (C) are means ± SE for at least 8 mice in the control (corn oil) and BA-treated groups and significant (p < 0.05) inhibition by BA is indicated (*).

Athymic nude mice bearing RKO cells as xenografts were treated with corn oil (control) or BA (25 mg/kg/d). Treatment with BA significantly decreased tumor growth and volume and this was accompanied by decreased tumor weights measured after sacrifice (Figures 6A and 6B). Lysates from control and BA-treated tumors were analyzed by western blot analysis for Sp1, Sp3 and Sp4 protein expression, and quantitated (relative to β-actin). The results showed that BA significantly decreased expression of Sp1, Sp3 and Sp4 (Figure 6C) and these results were consistent with comparable effects observed *in vitro *(Figure 2A).

## Discussion

The anticancer activity of BA initially showed high potency against melanoma in cell culture and animal models, and subsequent studies show the effectiveness of this compound against multiple tumor types [39,40,42]. The low *in vivo *toxicity of BA coupled with supporting *in vitro *and *in vivo *results suggest that this compound or some derivative has potential for clinical applications in cancer chemotherapy. However, BA is a highly lipophilic molecule with limited water solubility and this may decrease *in vivo *uptake of this compound; therefore, development of specialized formulations/carriers such as liposomes may help to enhance the *in vivo *efficacy of BA as an anticancer agent [43]. Previous studies in this laboratory showed that BA inhibits prostate cancer cell and tumor growth and this is accompanied by proteasome-dependent degradation of Sp1, Sp3 and Sp4 and several Sp-regulated pro-oncogenic gene products [20]. Several other anticancer agents including tolfenamic acid, curcumin, arsenic trioxide, a nitro-NSAID (GT-094), and two synthetic triterpenoid derivatives, CDDO-Me and CDODA-Me, also induce Sp downregulation in various cancer cell lines via proteasome-dependent and -independent pathways [19,21,33-38].

BA inhibits colon cancer cell growth and induces caspase-dependent PARP cleavage in RKO and SW480 colon cancer cells (Figure 1) and these results are consistent with other reports on the effects of BA on colon cancer cell lines [39,40,44-46]. Moreover, BA also inhibited tumor growth in athymic nude mice bearing RKO cells as xenografts (Figure 6). We observed that BA decreased expression of Sp1, Sp3 and Sp4 proteins in both RKO and SW480 colon cancer cells and tumors (Figures 2A and 6C) and this was accompanied by parallel decreases in survivin and VEGF (Figures 2A and 2B), and these results are comparable to those observed in LNCaP prostate and KU7 bladder cancer cells treated with BA [20,32]. Recent RNA interference studies show that p65 (NFκB subunit), EGFR, cyclin D1, and pituitary tumor transforming gene-1 (PTTG-1) are also Sp-regulated genes [32-35], and results in Figure 3C demonstrate that BA decreased expression of these gene products in RKO and SW480 cells. Moreover, knockdown of Sp1, Sp3 and Sp4 (in combination) in RKO colon cancer cells also decreased expression of EGFR, cyclin D1, p65 and PTTG-1, confirming the role of Sp transcription factors in regulating expression of these genes. These results are consistent with the induction of apoptosis by BA since many of these Sp-regulated genes are important for survival pathways.

Previous studies showed that BA-induced downregulation of Sp1, Sp3 and Sp4 was proteasome-dependent in LNCaP cells but proteasome-independent in KU7 bladder cancer cells [20,32]. Similar variability was observed in RKO and SW480 colon cancer cells (Figure 2D) where BA-induced downregulation of Sp proteins was proteasome-independent and -dependent, respectively. This demonstrates that, for BA and possibly other drugs that downregulate Sp1, Sp3, Sp4 and Sp-regulated genes, the pathways required for this response are variable and dependent not only on tumor type but also cell context within the same tumor. At least two of these pathways, namely induction of proteasome- and caspase-dependent degradation of Sp proteins, involve activation of post-transcriptional processes [20,21,37]; however, their mechanisms have not been determined and are currently being investigated in this laboratory.

We have previously reported that the synthetic triterpenoid CDODA-Me and the NO-NSAID GT-094 decrease Sp protein expression in SW480 and RKO colon cancer cells through a transcriptional repression pathway in which miR-27a is decreased and this results in the induction of ZBTB10, a transcriptional repressor [36,38]. BA decreased luciferase activity in RKO cells transfected with constructs containing several GC-rich promoter inserts (Figures 3B-D) and also decreased expression of miR-27a and induced expression of ZBTB10 in RKO cells (Figures 5A-C). Since overexpression of ZBTB10 and antisense-miR-27a also decreases expression of Sp1, Sp3, Sp4 and Sp-regulated genes in colon cancer cells [36], the mechanism of action of BA in RKO cells is linked to disruption of miR-27a:ZBTB10 as previously reported for CDODA-Me and GT-094 in colon cancer cells [36,38].

BA is known to be a mitochondriotoxic drug and decreases the mitochondrial membrane potential in several different cancer cell lines leading to induction of apoptosis [39,40,44] and BA also decreased MMP in RKO cells (Figure 4C). Previous studies have demonstrated that at least four agents that are mitochondriotoxic and induce ROS also downregulate Sp proteins; this effect is ROS-dependent and reversible with antioxidants or catalase, and compounds activating this pathway include arsenic trioxide (bladder), curcumin and CDDO-Me (pancreatic), and GT-094 (colon) [33,37,38]. Moreover, for GT-094 and CDDO-Me, the mechanism of ROS-dependent downregulation of Sp1, Sp3, and Sp4 involves disruption of miR-27a:ZBTB10 [33,38]. Results of this study show that BA also induced ROS-downregulated Sp1, Sp3, Sp4 and miR-27a and induced ZBTB10 in RKO cells, and all of these responses were significantly attenuated in cells cotreated with BA plus catalase (Figure 4). Moreover, catalase also reversed the growth inhibitory effects of BA (Figure 4C), further demonstrating the importance of ROS activation for the anticancer activity of this compound in RKO cells. In contrast to previous studies showing that CDODA-Me and GT-094 activated transcriptional repression of Sp proteins in both RKO and SW480 cells [33,36], BA induced transcriptional repression in RKO cells but activated the proteasome pathway for degradation of Sp proteins in SW480 cells. The mitochondrial or extra-mitochondrial origins of ROS in cancer cells treated with BA and other agents that downregulate Sp transcription factors is currently being investigated.

## Conclusions

In summary, we have shown that the anticancer activity of BA in colon cancer cells is due, in part, to downregulation of Sp1, Sp3, Sp4 and Sp-regulated prooncogenic gene products. The upstream mechanisms associated with decreased expression of Sp1, Sp3 and Sp4 are cell context-dependent and involves proteasome-dependent (SW480) and proteasome-independent (RKO) pathways. The response in RKO cells involves loss of MMP and induction of ROS as previously reported for BA in other studies [39,40] and this is coupled with ROS-dependent disruption of miR-27a:ZBTB10. BA also decreased luciferase activity in RKO cells transfected with a construct containing the -639 to +39 region of the miR-27a promoter, and we are currently examining the mechanisms associated with ROS-dependent effects on critical transcription factors interacting with the promoter and also the functional significance of ROS-dependent downregulation of miR-23a and miR-24-2 which form part of the miR-23a-miR-27a-miR24-2 cluster. These results coupled with several recent reports demonstrate potential clinical applications for BA and related compounds alone or in combination with other anticancer agents [47-49].

## Competing interests

The authors declare that they have no competing interests.

## Authors' contributions

SC carried out and supervised the *in vitro *studies on BA-induced downregulation of Sp proteins and Sp-regulated genes and also the RNA interference studies. SP carried out the *in vitro *studies on downregulation of Sp1, Sp3 and Sp4 and Sp-regulated genes. PL carried out some of the *in vitro *experiments including the studies on miR-27a:ZBTB10. SP carried out the *in vivo *study and analyzed the tumor tissue. SS carried out the experimental design and drafted the manuscript. All authors have read and approved the final manuscript.

## Pre-publication history

The pre-publication history for this paper can be accessed here:

http://www.biomedcentral.com/1471-2407/11/371/prepub
